# Evaluation of a novel surface-coating formulation with time-extended antimicrobial activity for healthcare environment disinfection

**DOI:** 10.1186/s13756-023-01341-w

**Published:** 2023-11-23

**Authors:** Roberto Bruno Maria Marano, Diana Merezhko, Keren Anat Resnick, Jacob Moran-Gilad, Yonatan Oster

**Affiliations:** 1 Department of Health Policy and Management, School of Public Health, Faculty of Health Sciences, Ben Gurion University of the Negev, Beer Sheva, Israel; 2 Clinical Microbiology Laboratory, Department of Clinical Microbiology and Infectious Diseases, Hadassah Hebrew University Medical Center, Jerusalem, Israel; 3 Faculty of Medicine, The Hebrew University of Jerusalem, Jerusalem, Israel

## Abstract

**Background:**

The importance of environmental contamination in the transmission of pathogens among hospitalized patients is universally recognized, and disinfection of surfaces is a widely accepted modality for reducing healthcare-associated infections. Nevertheless, hospital disinfection is still suboptimal. In this study, we evaluated the sustained effects of the novel formulation OxiLast™ which extends the antimicrobial effects of chlorine-based disinfectants.

**Methods:**

In an experimental lab phase, PVC surfaces were coated with OxiLast™ and then inoculated with representative Gram-positive and Gram-negative pathogenic bacteria. Cells were recovered at different contact times (5, 15, 30 min) to assess the reduction in bacterial counts compared to uncoated surfaces and also subject to various challenges to assess robustness. A similar methodology was then applied in an unoccupied hospital room to evaluate the sustained effect of OxiLast™ on high-touch surfaces.

**Results:**

OxiLast™ demonstrated notable activity against the range of bacterial strains tested with ≥ 4 log_10_ reduction in bacterial counts observed for up to seven days following one surface application, for various strains and contact times. Similar results were observed following challenges such as simulated abrasion of coated surfaces, organic contamination or successive inoculations. The results were confirmed in a simulated patient care environment.

**Conclusions:**

The addition of OxiLast™ to common chlorine-based disinfectants has shown a substantial and sustained reduction in bacterial pathogen counts for up to 7 days following one application. The consistent results in the laboratory and hospital are promising and should be tested in a real-life clinical scenario.

**Supplementary Information:**

The online version contains supplementary material available at 10.1186/s13756-023-01341-w.

## Introduction

Hospital-acquired infections (HAIs) are among the most frequent complications related to hospitalizations [1]. Many of these infections involve antibiotic-resistant organisms, further complicating the treatment and increasing morbidity, mortality, and costs [2]. Many factors contribute to such infections, especially in the cross-transmission of pathogens, including healthcare workers’ behavioral, environmental, and patient-related factors [3]. The healthcare environment, including design, materials used, cleaning methods and routines, equipment location, and patient density, were shown to influence the risk for HAIs [4]. Many strategies were developed to decrease the rate of cross-transmission of infections between patients, including hand hygiene [5], surveillance and early detection, and isolation of carriers of multi-drug-resistant organisms (MDROs) [6]. The key pathogens of concern are the ‘ESKAPE’ group, as designated by the World Health Organization, which includes *Enterococcus faecium, Staphylococcus aureus, Klebsiella pneumoniae, Acinetobacter baumannii, Pseudomonas aeruginosa*, and *Enterobacter* species [7].

The patient’s environment and hospital disinfection are important in HAIs prevention. Previous studies have shown that bacteria, including MDROs, colonize the immediate patient environment, which includes high-touch surfaces such as bed rails, food trays, and intravenous pumps, which are frequently touched by healthcare workers who can inadvertently transfer these pathogens to other patients [8]; a surface that looks clean to patients and hospital staff might indeed still serve as a cross-transmission point source. A recent meta-analysis has shown an odds ratio of 1.8 for being infected with an MDRO infection following exposure to a prior bed occupant with the same organism, demonstrating the importance of environmental contamination on MDROs acquisition [9]. The beneficial effect of disinfection in reducing environmental contamination and lowering the odds of HAIs was previously reported [10, 11]. Systematic reviews recently showed how increasing the frequency of disinfection could reduce the burden of pathogens on surfaces and thus HAIs, at least for certain pathogens [10], although standardized methods to assess outcomes are still missing [12]. Another systematic review pointed out a knowledge gap in the literature concerning how cleaning training of the hospital staff and disinfection activity and standards would impact HAI frequency [3]. Disinfection should be done only on clean surfaces because organic matter and existing biofilms, may reduce the antimicrobial activity of many disinfectants, and even fail to prevent subsequent new biofilm formation [13, 14].

The choice of a specific disinfecting agent is related to its antimicrobial activity but also to its ease of use, safety, and cost. Common disinfectants recommended for use in healthcare are chlorine-based disinfectants, quaternary ammonium compounds, and hydrogen peroxide. However, effective environmental disinfection is often limited by the ability to enforce institutional cleaning regimens, perform cleaning in a timely manner while minimizing the disruption of the hospital’s workflow and optimize the use of hospital rooms and facilities and assess its effectiveness [15]. Due to these practical limitations, the US Centers for Disease Control and Prevention (CDC) based its guidelines on the frequency of environmental disinfection and cleaning on a risk-based approach [16], where the probability of environmental contamination in every setting is assessed along with the potential for exposure and the vulnerability of exposed patients to create specific recommendations for different hospital settings. In most hospital units, the immediate patient environment, including high-touch surfaces, is cleaned and disinfected once daily, but this may occur less frequently in real-life conditions, for example due to reduction in hospital staff during weekends and holidays. These unavoidable time-gaps between disinfection cycles leave a periodic neglect of disinfection of high-touch surfaces and create an opportunity for contaminated surfaces to further contribute to the transmission of infection. This happens since no standard disinfection method currently provides sufficient residual activity for contamination prevention until the next disinfection cycle. A disinfectant technology that maintains its antimicrobial activity “prospectively” until its next application is missing for high-touch surfaces like the patients’ surroundings.

To overcome this gap, novel disinfection technologies are needed. OxiLast™ is a newly patented coating material that can be added to a chlorine-based disinfecting solution and create an invisible temporary film that preserves and extends the bactericidal capacity of the added chlorine during prolonged gaps between disinfection cycles to attenuate or even eliminate sustained and successive microbial contaminations. The coating itself does not have independent antimicrobial properties. In this study, we examined for the first time the effect of chlorine-based disinfection combined with OxiLast™ in different in-vitro conditions and in a model unoccupied hospital room using standardized testing methods to establish its effectiveness and potential usability for disinfection of the hospital environment.

## Materials and methods

### Properties and chemistry of the OxiLast™ formulation

OxiLast™ (a product of Bio-fence, Israel) is an innovative dilutable water-based formulation designed to form a thin and transparent film that holds a patented compound (patent number WO2021/245,663 A4). The formulation contains the OxiLast™ additive and a film-forming polymer. The additive is the patented new compound obtained in powder form that, due to its chemical structure (rich-nitrogen core), has a high affinity to attract a negatively-charge molecule such as OCl^−^ (originated from dissolving the Sodium Dichloroisocyanurate, NaDCC in water). In that, OxiLast™ is not a standalone disinfectant but rather an adjuvant to chlorine. It is worth noting that OxiLast™ does not alter the oxidative power or efficient killing properties of active chlorine. This is because the stabilization of chlorine by OxiLast™ is solely accomplished through hydrogen bonds and electrostatic interactions, which delay the release of chlorine, overcoming the limitation of its short lifespan. When added to water and chlorine, no new chemical compounds are formed, and the fundamental chemistry of chlorine remains unaltered.

The film formation is achieved by including a distinctive water-soluble polymer that possesses both aliphatic and polar groups, enabling it to effectively adhere to a wide range of surfaces, such as plastic, metal, wood and resin-coated materials. Consequently, this formulation can be applied to nearly any surface found in dry environments, particularly in patient rooms. The adhesion of the resulting film to each surface is sufficiently robust to provide protection, yet it remains easily removable with water and soap. OxiLast™ is metal-free and therefore, no metal salts are released. The additive and polymer are classified as non-hazardous according to the Globally Harmonized System of Classification and Labelling of Chemicals (GHS). Risk assessment concerning dermal, oral and inhalational exposure to the chlorine available while using the product concluded that no excess risk is foreseen to professional users applying the product and patients and general public present.

### Study plan

The overall efficacy of the OxiLast™ formulation was evaluated by inoculating coated and non-coated surfaces with standardized inocula (defined amounts of cells) of selected model Gram-positive and Gram-negative bacterial pathogens relevant to HAIs under different conditions and contact times (CT) and performing live counts on solid media. Initially, a laboratory-based experiment was devised and carried out to test the general antimicrobial efficacy of the formulation, for which most of the procedures were based on the ISO 22196:2011: ‘Measurement of antibacterial activity on plastics and other non-porous surfaces’ [12] with certain modifications, where the formulation was tested as a function of CT with defined target bacterial inocula. Three additional testing conditions were evaluated in the laboratory setting (i.e. challenges). These included inoculation with organic load (simulating contaminated body fluids), mechanical abrasion of the coating (simulating abrasion during everyday activities), and successive loadings (simulating repeated hospital exposures); the first two were based on the BSI-PAS-2424 2014 guidelines [17], with reported modifications. Subsequently, a modeled environmental experiment was conducted in a real unoccupied hospital room to test the antimicrobial effectiveness when applied onto surfaces considered hot spots for pathogen circulation in hospitals while comparing with the hospital routine disinfection procedure and the presence of organic load simulants. All modifications to standard methods are listed in Tables 1 and 2. A schematic view of the study plan is provided in the Supplementary Material (Figure S1).

**Table 1:** Description of modifications applied to the original ISO22196-2011 protocol

Variable	Original ISO method	Applied modification	Rationale
Bacterial strains	*Staphylococcus aureus* *Escherichia coli*	Methicillin-resistant *Staphylococcus aureus* (MRSA) ATCC 33591 Vancomycin-resistant *Enterococcus* (VRE) ATCC 51299 *Pseudomonas aeruginosa* ATCC 9027 *Acinetobacter baumannii* ATCC 19606 Carbapenem-resistant Enterobacterales *– Klebsiella pneumoniae* ATCC BAA-1705.	Gram-positive and Gram-negative representatives of the ESKAPE bacteria, which are commonly problematic in hospital-acquired infections and associated with multi-drug resistance profiles (7). Among the tested strains, *P. aeruginosa* and *A. baumannii* are not carbapenem-resistant.
Contact time	24 h	5 min, 15 min, 30 min.	The OxiLast™ formulation is devised to act prospectively following its application and within a short time.
Inocula concentration of tests and controls	A target concentration of 1–4 × 10^5^ cells in 0.4 ml used for inoculation onto the substrate surfaces.	A target concentration of 1–4 × 10^7^ or 1–4 × 10^6^ cells in 0.4 ml was used for inoculation onto the substrate surfaces.	Higher CFU counts in the inocula were used to prove efficacy at high inoculation regimens and to measure a desired 4 log_10_ reduction within the limit of detection of the procedure used.
Test surface and cover film sizes	Standard size of the cover film shall be a square of (40 ± 2) mm ×(40 ± 2) mm for the 50 mm ×50 mm test specimen.	Cover films used were square of (80 ± 2) mm ×(80 ± 2) mm for the 100 mm ×100 mm test substrate.	A larger surface, with the same volume was chosen to maximize contact between the bacterial cells and the OxiLast™ coating.
Coating age	Not specified.	Freshly prepared, 24 h, 48 h, 72 h, 5 days, 7 days.	Because the OxiLast™ formulation is devised to exert its antimicrobial properties prospectively, this effect was tested over the indicated coating ages.
Antibacterial neutralizer solution	Soybean casein digest broth with lecithin and polyoxyethylene sorbitan monooleate broth (SCDLP broth)	Sodium-Thiosulfate 1 mg/ml in 0.85% (w/v) NaCl.	Sodium-thiosulfate is used for chlorine species neutralization in antimicrobial testing (18), and the ISO standard allows the use of a neutralizer of choice upon a provided toxicity test (conducted).
Neutralizer solution volume used	10 ml.	5 ml.	A lower resuspending volume was used to minimize dilution effects due to the lower volume used in plating (i.e. 200 µl instead of 1 ml).
Recovery of bacteria from test specimens	Mechanical methods such as stomaching, vortexing or sonicating	Scraping of PET covers and PVC substrate slides with sterile scrapers 20 s in two directions after applying the neutralizer solution.	This method allowed for > 99% recovery efficiency of viable cells in control slides.
Determining the viable bacteria count	Stirring of 1 ml of recovered neutralizer solution (or serial dilutions of it) onto pre-solidified PCA plate together with 15 ml of the same melted culture medium	Plating of 0.2 ml of recovered neutralizer solution (or serial dilutions of it) onto TSA plates or otherwise specified selective agar media.	Because commercial ready-to-use plates were used, the maximum volume that could be practically plated was 200 µl.

**Table 2:** Description of modifications applied to the original BSI-PAS-2424-2014 protocol

Parameter	Original BSI-PAS-2424-2015 method	Applied modification	Rationale
Dry abrasion test cycles	Three cycles of dry&wet abrasion, with intermittent inoculations and disinfectant application in between cycles.	Three dry abrasion cycles (with each cycle including one forward and one backward motion) were applied on a coated surface before the bacterial inoculation	While the original test was devised to test antimicrobial properties of disinfectants in both dry and wet abrasions, our test was conceived for surfaces exposed to dry hand-touch or dry cloth abrasions only. Thus the test was conducted by exposing the coating to all the dry abrasions at once and then inoculating with the test bacteria.
Resuspension of viable cells after the abrasion test for enumeration.	Immersion of the tested surface into 10 ml of the neutralizer solution.	5ml of neutralizer solution (as per Table 1) and scraping of PET covers and PVC substrate slides with sterile scrapers 20 s in two directions after the application of the neutralizer solution.	The same cells resuspension method was used for all tests due to established high recovery efficiency and to minimize technical variations.

### Bacterial strains used

The following ATCC reference strains were used and tested, representing both Gram-positive and Gram-negative pathogens of medical importance: methicillin-resistant *Staphylococcus aureus* (MRSA) ATCC 33591, vancomycin-resistant *Enterococcus faecalis* (VRE) ATCC 51299, carbapenem-resistant *Klebsiella pneumoniae* ATCC BAA-1705, *Pseudomonas aeruginosa* ATCC 9027, *Acinetobacter baumannii* ATCC 19606. The MRSA, VRE and *Klebsiella* strains represent multi-resistant isolates while *P. aeruginosa* and *A. baumannii* strains used were relatively susceptible and not carbapenem-resistant. For testing steps in which only two species were included, a Gram-positive and a Gram-negative species were always included.

Additional carbapenem-resistant *K. pneumoniae* (*bla*_NDM_-producing) and MRSA isolates recovered at the Clinical Micrbiology Laboratory of the Hadassah Hebrew University Medical Center from routine cultures were used in the modeled environmental experiment; these strains were identified by MALDI-TOF MS (VITEK MS, bioMeriuex, Marcy l’Etoile, France) and their susceptibility was confirmed using the VITEK2 (bioMerieux) and in house PCR testing per institutional microbiology protocols.

### Formulation preparation and application

The OxiLast™ ready-to-use (RTU) formulation was prepared by dissolving 46.7 g of the concentrated formulation into 286.3 g of double distilled water and subsequently adding one tablet of sodium dichloroisocyanurate (NaDCC) (Klorkleen®, effervescent tablet; Medentech, Ireland), for a final concentration of 3000 ppm (i.e. 3 g/l) of active chlorine. For the laboratory phase experiments, the formulation was applied onto PVC slides with two cycles of spraying three times with a nebulizer bottle and subsequent dispersion of the liquid layer with a clean cloth moisturized with the same formulation to obtain a visibly uniform coating. Dry-coated slides were then stored at room temperature until use and transferred to sterile 12 × 12 × 1.7 cm^3^ petri dishes (Greiner Bio One, Kremsmünster, Austria) prior to use. For the modeled environmental experiment, a dry microfiber wipe (29 × 29 cm^2^) was fully immersed and moisturized within 60 g of the formulation and applied slowly in an S-shape motion, covering an area of 0.4 m^2^. In the same modeled environmental experiment, the routine KlorKleen® suspension was made by dissolving 1 tabled of the product to 1 L of water and used as above. Finally, the coated or disinfected surfaces described above were left to dry and remained untouched for a minimum one hour or until use (Table 3).

### Bacterial inocula preparation

Bacterial suspensions for inocula were freshly prepared before use according to ISO 22196 with minor modifications by resuspending a loopful of 16–24 h old bacterial culture from tryptic-soy agar (TSA) (Hylabs, Rehovot, Israel) in a small volume of sterile diluted nutrient broth (NB) (Merck, Darmstadt, Germany) (made with a 1:500 dilution of NB in sterile water). Subsequently, more of such diluted NB was added to achieve a McFarland 0.5 density measured using a Densicheck Plus reader (BioMerieux, Marcy-l’Étoile, France), equivalent to a ~ 10^8^ colony forming units (CFU) per ml. The initial 0.5 McFarland stock was further diluted (using the diluted NB solution) to meet the final target inocula described below. Bacterial stock suspensions were confirmed by live counts, by plating aliquots of appropriate serial dilutions made in 0.85% (w/v) sterile NaCl solution on TSA plates and incubating for 24 h at 37 °C.

### Laboratory phase experiment

For the laboratory phase experiments, clean 10 × 10 cm^2^ non-smooth PVC slides and 8 × 8 cm^2^ PET cover films were disinfected by immersion in 70% (v/v) ethanol overnight and air-dried before use. Four experimental tests were conducted in the laboratory phase experiment, as described below. Different arrays of coating ages, CTs, or CFU loads were used for each test, as summarized in Table 3. Coating ages spanned from freshly prepared (i.e. 1 h before testing) to one, two, three, five, and seven days-old coatings. The CTs of the tested inocula applied onto OxiLast-coated surfaces varied between 5, 15, or 30 min. The chosen testing inocula were in a range that is equivalent to 0.6 log_10_ range in line with the ISO 22,196, being 1-4 × 10^7^ and 1-4 × 10^6^ CFU for most cases (see below). For each of the below-described experiments, the sterility of the tested PVC slides and films from every batch, as well as the procedure itself, were ensured by testing a negative control (i.e. no bacteria used in the procedure).

(i) General antimicrobial efficacy testing

The basic antimicrobial activity was evaluated based on ISO 22196 and involved all five ATCC reference bacterial strains. For each defined CT, 1-4 × 10^7^ CFU were inoculated in triplicate onto OxiLast™ coated and single control (non-coated) PVC slides by transferring 400 µl of fresh 0.5 McFarland suspension and covering them with the PET cover slides for uniform distribution over an 8 × 8 cm^2^ area. Where a 1-4 × 10^6^ CFU inoculum was used, 400 µl from a 1:10 dilution of the 0.5 McFarland suspension was used.

(ii) Organic load challenge

Based on BSI-PAS2424, a suspension of 1-4 × 10^7^ CFU in a 3 mg/ml solution was prepared by mixing equal volumes of a sterile 6 mg/l bovine serum albumin (BSA) fraction V (Merck, Darmstadt, Germany) solution and a 1:5 dilution of the 0.5 McFarland suspension. For each defined CT, 1-4 × 10^6^ CFU were inoculated by transferring 400 µl of such suspension in triplicate onto OxiLast™ coated and single control (non-coated) PVC slides and covering them with the PET cover slides as above. Tested species included the above described reference strains MRSA ATCC 33591 and *P. aeruginosa* ATCC 9027 as representative species of biofilm-producing Gram-positive and Gram-negative bacteria.

(iii) Successive loading test

Two different CFU load regimes were used to inoculate the same surface at a defined CT of 15 min each. Triplicate OxiLast-coated test PVC slides and one control were initially inoculated with 360 µl of a 1:10 dilution of the 0.5 McFarland suspension (equivalent to 1-4 × 10^6^ CFU); subsequently, 40 µl of the same suspension (equivalent to 1-4 × 10^5^ CFU) were used to re-inoculate the same surface. This approach would ensure a compromise between a tolerable deviation of 10% from the established 1-4 × 10^6^ CFU inoculation, and the final volume tested on a single PVC slide. The reference strains MRSA ATCC 33591 and *P. aeruginosa* ATCC 9027 were tested in this experiment, as above.

(iv) Abrasion test

OxiLast-coated PVC slides were challenged with an adapted mechanical abrasion test based on the BSI-PAS2424. An apparatus was assembled with a flat base of 10 × 10 cm topped with a weight such that the neat apparatus’ weight was 2,185 g and the exerted weight per cm^2^ at the base was 21.85 g. A polypropylene wipe was wrapped at the apparatus’ base and OxiLast-coated and control slides were subjected to three cycles of a forward and backward motion application of the polypropylene wipe with neat pressure applied only by the apparatus’ weight onto the whole area of the coated surfaces, as per BSI-PAS2424. Finally, triplicate OxiLast-coated abrased slides were tested along with single non-coated abrased controls by inoculation with a 1-4 × 10^6^ CFU per surface and processed as per the general testing and with 30 min CT only. The reference strains MRSA ATCC 33591 and *P. aeruginosa* ATCC 9027 were tested in this experiment, as above.

### Bacterial cell recovery and enumeration in the laboratory phase

At the end of each defined CT, the PET cover films were lifted, and the inocula were washed with 5 ml of sterile 1 mg/ml sodium thiosulfate solution (Merck, Darmstadt, Germany) to neutralize and deactivate the active chlorine [18]. Inoculated surfaces and PET covers were then thoroughly scratched with 20 backward and forward motions in four directions with a sterile cell spreader to resuspend cells. Aliquots (200 µl) of resuspended cells and related serial 10-fold dilutions in 0.85% NaCl were then plated in duplicates on TSA plates and incubated for 24 h at 37 °C and colonies enumerated; plates were then reincubated for 24 additional hours, and colonies recounted for variations check. A toxicity test was conducted as per ISO 22196 to exclude adverse effects of the thiosulfate solution on the tested bacteria strains (data not shown).

### Modeled environmental experiment

In the modeled environmental experiment, a non-occupied standard hospital room made available for the experiment at the Hadassah Medical Center was used. The setting included two patient beds (with the semi-smooth PVC bedrail surfaces being tested), two cabinets with food trays (also PVC and semi-smooth), and a long windowsill (smooth resin-coated surface), representing three types of ‘high touch’ surfaces. The surfaces were inoculated with defined amounts of CFU of different strains, simulating accidental contamination of cleaned surfaces treated with the routine hospital disinfectant as the comparator, or OxiLast™, and further tested with or without an organic matrix simulant.

Tested surfaces were initially cleaned of any dust and dirt by employing a commonly used detergent-based product, and then chlorine-based disinfectant used at the hospital (Klorkleen®, 1000 ppm active chlorine) as per the hospital’s standard procedures and finally wiped with 70% (v/v) ethanol to ensure maximized disinfection, prior to use. Surfaces were then tested as such (controls), or after disinfection with the standard chlorine-based disinfectant or coating with OxiLast™. The tested surfaces in the modeled environmental experiment included selected areas of 4 × 5 cm^2^. Fresh bacterial suspensions of 1-4 × 10^7^ CFU/ml in 5 ml volume were prepared using 0.5 ml of a McFarland 0.5 suspension prepared as above, 3.5 ml of sterile NaCl 0.85%, and 1 ml tryptic-soy broth (TSB) (Merck, Darmstadt, Germany). Selected areas from all tested conditions (Table 4) were inoculated with 1-4 × 10^6^ CFU applied as 20 droplets of 5 µl from the above suspension, as previously described [19], and let dry at room temperature for precisely 1 h. Tested species included the reference strains MRSA ATCC 33591 and *K. pneumoniae* ATCC BAA-170*5*, for all tests, and the MRSA and *K. pneumoniae* clinical isolates in indicated tests (see below); the *K. pneumoniae* strains were chosen because carbapenemase-producing Enterobacterales (CPE), including *K. pneumoniae* species, are endemic in globally as well as in Israeli hospitals [9] and preventing the spread of these species are of particular interest for the infection and prevention control units of hospitals. For each tested condition described below, a comparison was made between two test surfaces and one negative control (no coating or treatment).

Four experimental conditions were tested in this modeled environmental experiment, as described below and detailed in Table 4.

(i) General antimicrobial efficacy testing

The antimicrobial efficacy of the formulation was tested on bedrails, cabinets, and windowsill for both ATCC reference strains on OxiLast™ coatings up to seven days old.

(ii) Comparison with hospital routine disinfectant

The possible residual disinfection capacity of the routine hospital disinfectant solution was evaluated on bedrails, cabinets, and windowsills for both ATCC reference strains on surfaces freshly disinfected, as per hospital routine, or after one day only. No residual effect was expected.

(iii) Extended testing on clinical isolates

To reinforce the results from (i) the antimicrobial efficacy of the OxiLast™ coating was tested with two routine hospital isolates of MRSA and *K. pneumoniae*, which were tested on fresh (day 0), and one and three-day-old coatings; tested surfaces included bedrails and cabinets.

(iv) Organic compounds

The OxiLast™ formulation was further tested in the presence of an organic compound mixture to assess any potential interference of organic material with the chlorine-based antimicrobial activity of the formulation. In this case, the bacterial suspension as above was spiked with additional artificial test soil (ATS) (Healthmark Industries Company, Inc., Frasier MI) to a final concentration of 3 mg/ml to simulate organic content from body fluids (e.g. proteins, hemoglobin, carbohydrates, mucin, cellulose and lipids, not including defibrinated blood) as previously described [20]. The tested coating ages included fresh (day 0), and one and three days old coatings; tested surfaces included bed rails and cabinets.

### Viable bacterial cell recovery and enumeration in the modeled environmental experiment

Dried inoculated areas were processed as previously described [19] by using a combination of a wet and a dry flocked nylon swabs to retrieve bacterial cells; swabs were then stored in 2.5 ml of commercial bacterial storing Swab Rinse Kit solution (SRK, Copan Diagnostic, Murrieta, USA) and kept at 4 °C for 2–3 h until processed. Sample tubes with swabs were thoroughly vortexed for 15 s and direct and serial 10-fold dilutions 0.85% NaCl aliquots (100 µl) were plated. For each species and each dilution, plating onto two agar plates was done in parallel: a rich non-selective medium (TSA) for maximizing bacterial growth from the OxiLast-coated surfaces and a selective or differential medium to assist with colony counts in case of substantial contamination. These included mannitol salt agar for MRSA, and CHROMagar orientation for *K. pneumoniae* (all from Hylabs, Rehovot, Israel). Plates were incubated as for the laboratory phase experiment and colonies were enumerated after 24 and 48 h. Growth was evaluated for comparison and differentiation of sporadic off-target colonies on the cultures. Whenever a substantial discrepancy arose (i.e. >20% CFU counts between the two technical replicates), this was always to the detriment of the TSA plate (non-selective) and the count on that plate was discarded.

### Viable bacterial cell estimation, recovery efficiency, and antimicrobial efficacy

The CFU on all inoculated surfaces at each endpoint of CT for both tests and controls were estimated by means of the liquid bacterial resuspensions in the neutralizer solution after testing, according to the formulas below:

$$Nt=\left(\frac{Np\times df}{Vp}\right)\times Vf$$

Where ‘Nt’ is the total number of cells on the tested surface, ‘Np’ is the average colony number on two replicate plates from a given dilution, ‘df’ is the dilution factor, ‘Vp’ is the volume plated in ml, and ‘Vf’ is the final suspension volume in ml (5 ml for the laboratory phase experiment and 2.5 ml for the modeled environmental experiment). To maximize stringency during the laboratory phase testing, in compliance with ISO 22196, whenever no colonies were observed from direct plating of the neutralizer solution, the reported number per ml of Np was 5, corresponding to the inferred limit of quantification (LOQ) of 1 CFU per volume plated of the resuspension in the neutralizer solution (0.2 ml).

The recovery efficiency of cells with the two described methods was calibrated on the controls as the ratio between: (i) the Nt recovered from the control surfaces and (ii) the CFU number per inoculum on the same controls; the latter was calculated (based on volume used) from the actual cell count per ml of the McFarland 0.5 suspensions used in each experiment to generate the inocula.

The antimicrobial efficacy was estimated in each tested condition as:

$$R=\frac{{\sum }_{0}^{n}\left(Log Nt Control-Log Nt Test n\right)}{n\,tests}$$

Where ‘R’ is the antimicrobial efficacy expressed as the average log_10_ reduction of the inocula from **n** test surfaces with respect their respective single controls.

A summary of all CFU counts retrieved from all controls in all the described experiments is provided in Supplementary Table S1.

### Endpoints

The antimicrobial efficacy of the OxiLast™ formulation was considered adequate when a ≥ 4 log_10_ reduction was evident. The minimum expected activity was set at ≥ 3 log_10_ reduction.

**Table 3:** Conditions used for the laboratory phase experiment with OxiLast™ coating

Objective	CT (minutes)	Coating (days)	Tested strains	Tested surfaces	CFU inocula used
General efficacy testing	5, 15, 30	0, 1, 2, 3, 5, 7	*P. aeruginosa* ATCC 9027	64 cm^2^ PVC; 3 tests 1 control	10^7^ for all CT 15 and 30 min, and CT 5 min at days 0 to 2. 10^6^ for CT 5 min on days 3, 5, and 7
			MRSA ATCC 33591	64 cm^2^ PVC; 3 tests 1 control	10^7^ for all CT 30 min, and for CT 15 min at day 0. 10^6^ for CT 5 min at day 0 and all CT 15 and 30 min on days 1 to 7
			* A. baumannii* ATCC 19606	64 cm^2^ PVC; 3 tests 1 control	10^7^ for all
			*K. pneumoniae* ATCC BAA-1705	64 cm^2^ PVC; 3 tests 1 control	10^7^ for all CT 15 and 30 min, and CT 5 min on days 0 to 2. 10^6^ on CT 5 min on days 3 to 7
			VRE ATCC 51299	64 cm^2^ PVC; 3 tests 1 control	10^6^ for all
Organic load	15, 30	0, 1, 2, 3, 5, 7	*P. aeruginosa* ATCC 9027	64 cm^2^ PVC; 3 tests 1 control	10^6^ for all
			MRSA ATCC 33591	64 cm^2^ PVC; 3 tests 1 control	
Successive loading	15 + 15	0, 1, 2, 3, 5, 7	*P. aeruginosa* ATCC 9027	64 cm^2^ PVC; 3 tests 1 control	10^6^ first inoculum and 10^5^ second inoculum
			MRSA ATCC 33591	64 cm^2^ PVC; 3 tests 1 control	
Mechanical abrasion	30	0	*P. aeruginosa* ATCC 9027	64 cm^2^ PVC; 3 tests 1 control	10^6^ for all
			MRSA ATCC 33591	64 cm^2^ PVC; 3 tests 1 control	

**Table 4:** Conditions used in the modeled environmental experiment testing

Objective	Contact time (minutes)	Coating age (days)	Tested strains	Tested surfaces	CFU inocula used	Product tested
Efficacy testing on three types of OxiLast-coated surfaces	60	0, 1, 2, 3, 5*, 6**, 7	MRSA ATCC 33591, *K. pneumoniae* ATCC BAA-1705	20 cm^2^ on bedrail, cabinet, and windowsill; 2 tests 1 control each	10^6^ for all	OxiLast™
Efficacy testing of routine chlorine-based product (1000 ppm) on three types of surfaces.	60	0, 1 (repeated twice each)	MRSA ATCC 33591, *K. pneumoniae* ATCC BAA-1705	20 cm^2^ on bedrail, cabinet, and windowsill; 2 tests 1 control each	10^6^ for all	Klorkleen®
Efficacy testing of OxiLast™ against clinical isolates	60	0, 1, 3	Clinical isolates of MRSA and *K. pneumoniae*	20 cm^2^ on bedrail and cabinet; 2 tests 1 control each	10^6^ for all	OxiLast™
Efficacy testing of OxiLast™ in presence of organic compounds***	60	0, 1, 3	MRSA ATCC 33,591, *K. pneumoniae* ATCC BAA-1705	20 cm^2^ on bedrail and cabinet; 2 tests 1 control each	10^6^ for all	OxiLast™

* Bedrails and windowsill only; ** Bedrails and cabinets only; *** ATS (artificial test soil 3 mg/ml). Each control consisted of an untreated surface with the same inoculation regime of the test areas

## Results

### General testing in the laboratory phase experiment

**Fig. 1:**
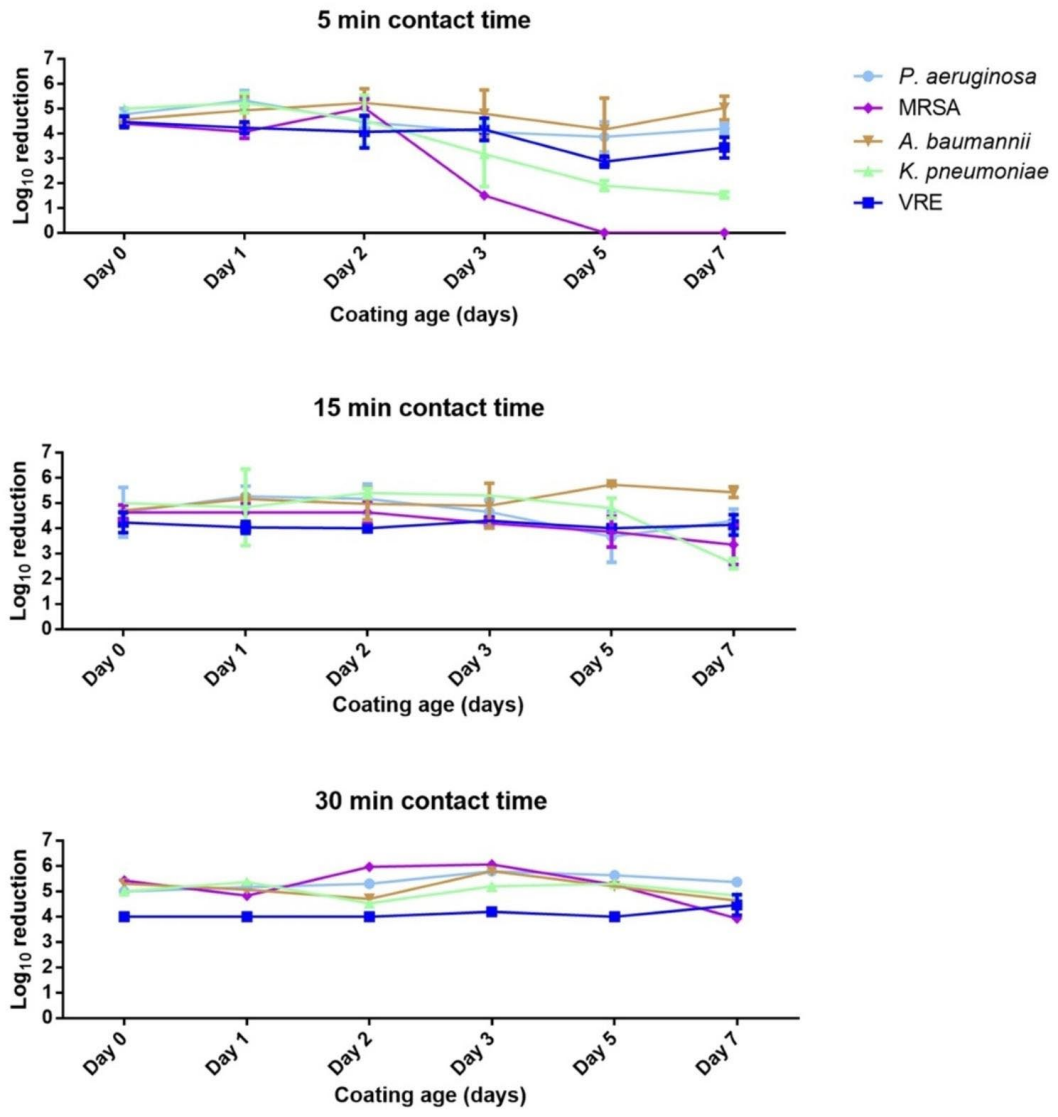
Antimicrobial capacity of the tested formulation expressed as log_10_ reduction of five tested ATCC reference bacterial strains inoculated onto Oxilast-coated PVC surfaces at various coating ages (days) and kept for 5, 15, 30 min contact time. Error bars, where visible, indicate standard deviation

In all control specimens, the CFU recovery was as expected (equivalent to a 10^6^ or 10^7^ CFU inoculum in the respective cases), with a standard deviation ≤ 0.2 log_10_ for each strain, except for *P. aeruginosa* (0.26 log_10_), showing the consistency of the used method. The calculated recovery efficiency of the method based on controls was > 99% in nearly all cases (except for two single replicate cases with 80% and 70% recovery of *A. baumannii* on days 2 and 5, respectively). A full depiction of the general testing results is presented in Fig. 1.

In this experimental setup, the three tested CTs differentially impacted the efficacy of the tested formulation. At 10^7^ CFU inoculation, the 5 min CT proved to be fully effective with ≥ 4 log_10_ reduction on *A. baumannii* up to day 7, *P. aeruginosa* and *K. pneumoniae* up to day 2, while At 10^6^ CFU inocula, 5 min CT was almost fully effective on *P. aeruginosa* up to days 3, 5, and 7. Against the Gram-positives, the formualtion was effective up to day 3 on VRE, and up to day 2 on MRSA.

When the CT was increased to 15 min, at 10^7^ CFU, a > 4 log_10_ reduction was observed on *P. aeruginosa*, *A. baumannii* up to day 7, and on *K. pneumoniae* up to day 5, and MRSA at day 0 only. At 10^6^ CFU, a 15 min CT was fully effective up to day 3 for MRSA with improved efficacy until day 5 with 3.9 log_10_ reduction, and fully effective on VRE up to day 7.

Finally, with a 30 min CT, at 10^7^ CFU, the formulation was fully effective on *P. aeruginosa, K. pneumoniae* and *A. baumannii* until day 7, and fully effective on MRSA up to day 5. At 10^6^ CFU, the 30 min CT was fully effective on MRSA and VRE (both Gram-positive) up to day 7. In some cases, minor inconsistencies were observed, like for *P. aeruginosa* with < 4 log_10_ reduction on day 5 at 5- and 15-minute CT but fully efficient at both CT on day 7. This could be due to inevitable variations in coating applications.

### Additional tests in the laboratory phase

Following the general testing results, two bacterial species only, *P. aeruginosa* and MRSA, were further selected to test the OxiLast™ coatings with conditions aimed at mimicking real-life use scenarios (Table 3). For each species, two strains were used, a reference ATCC strain as described above and a hospital clinical isolate, isolated in the same hospital where testing was conducted. The recovery from control specimens was as expected for all tested challenges.

**Fig. 2:**
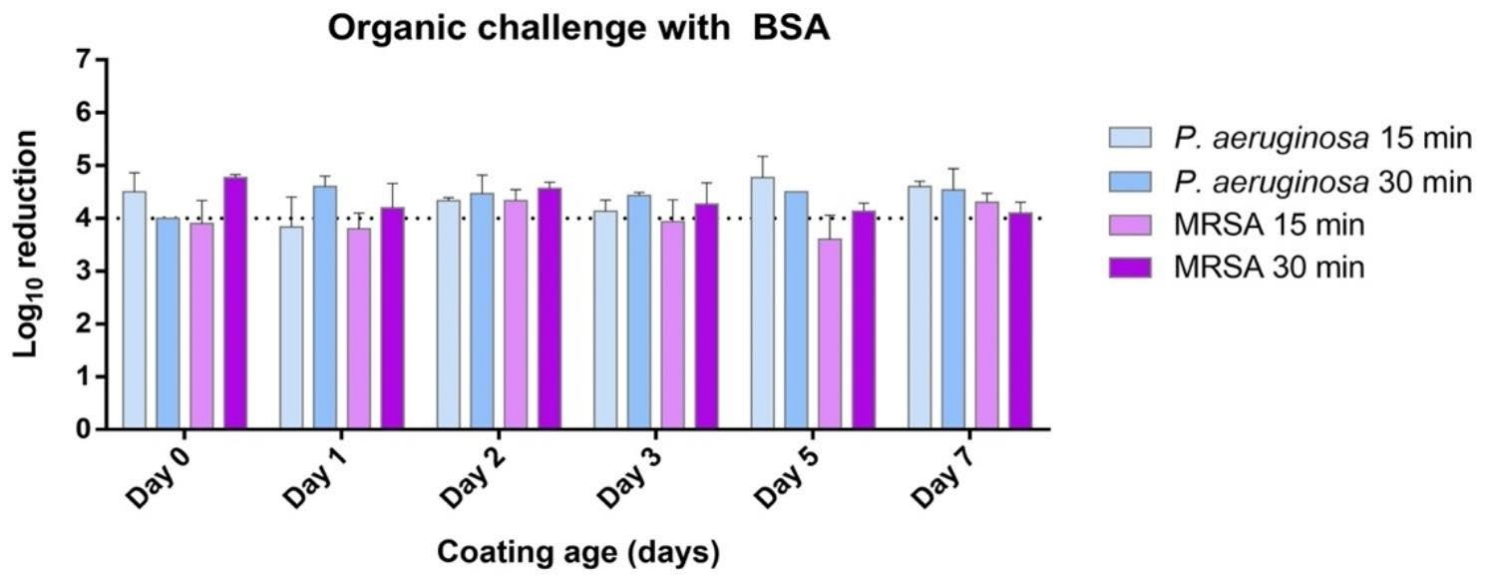
Laboratory phase: antimicrobial capacity of the tested formulation expressed as log_10_ reduction of two tested drug-resistant ATCC reference bacterial strains inoculated onto OxiLast-coated PCV surfaces at various coating ages (days) and kept for 15 or 30 min contact time in presence of bovine serum albumin (BSA) 3 mg/ml. Error bars indicate standard deviation

**Fig. 3:**
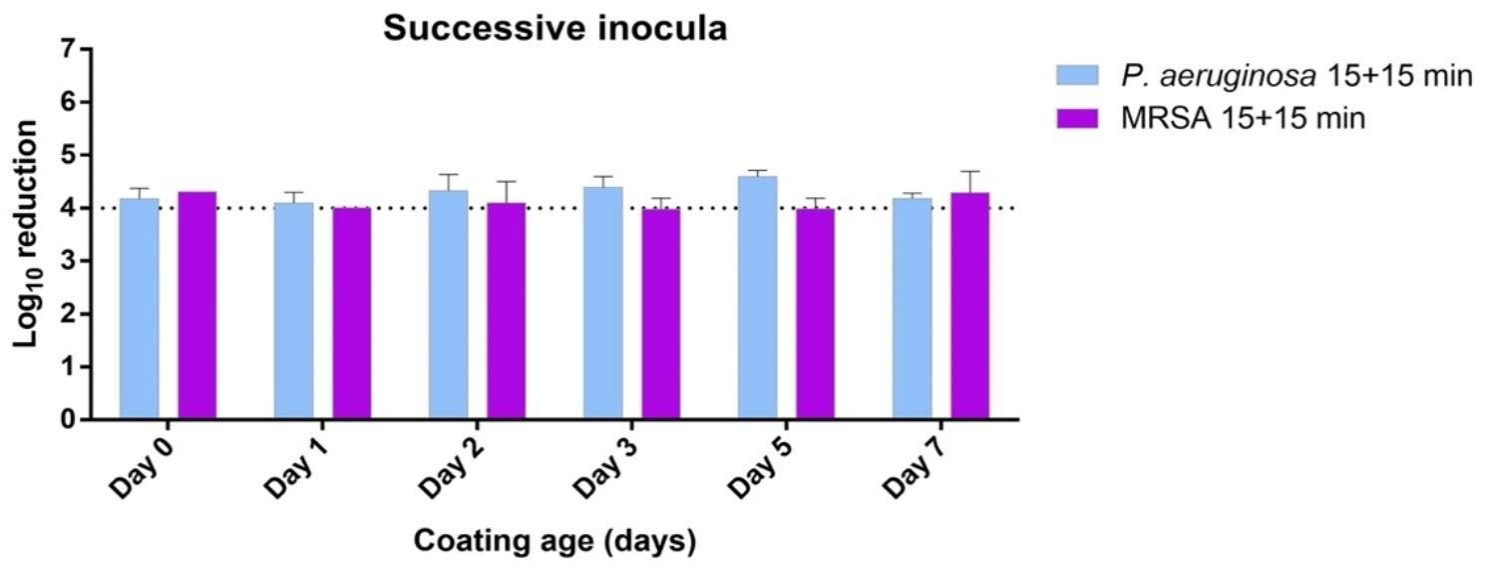
Laboratory phase: antimicrobial capacity of the tested formulation expressed as log_10_ reduction of two tested drug-resistant ATCC reference bacterial strains inoculated onto OxiLast-coated PCV surfaces at various coating ages (days). Inoculation was made with an initial load of 10^6^ CFU regime kept for 15 min contact time, followed by an additional load of 10^5^ CFU regime for an additional 15 min. Error bars indicate standard deviation

**Fig. 4:**
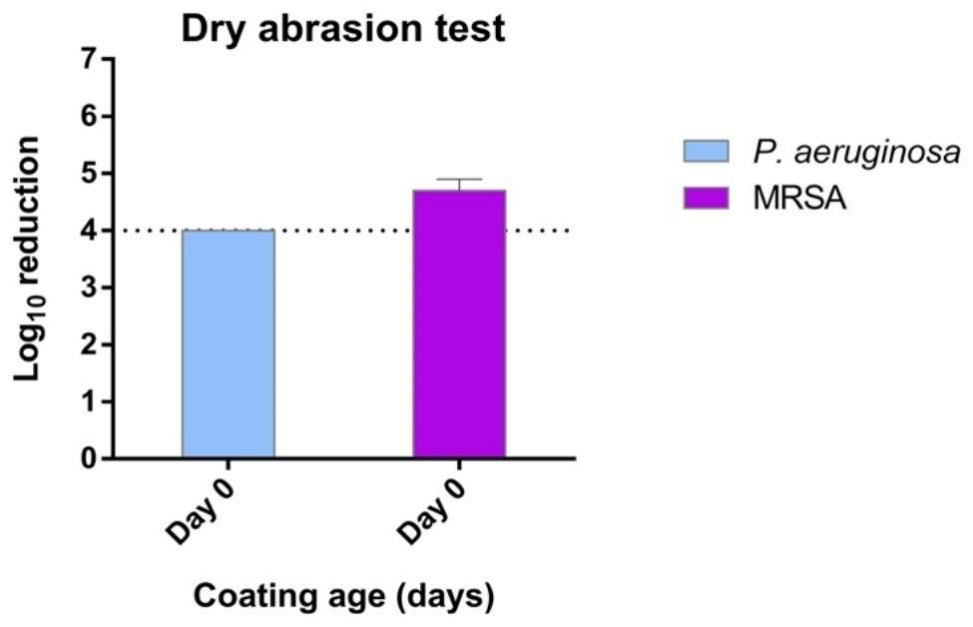
Laboratory phase: antimicrobial capacity of the tested formulation expressed as log_10_ reduction of two tested drug-resistant ATCC reference bacterial strains inoculated onto OxiLast-coated PCV surfaces (day 0) that underwent three mechanical dry abrasions prior to inoculation and testing. Inocula were kept for a 30 min contact time. Error bars indicate standard deviation

In the organic load challenge, the tested formulation systematically provided a ≥ 4 log_10_ average reduction in all cases at both 15- and 30-minutes CT, with 30 min CT, although some variation was observed for the 15-minute CT in particular with MRSA, seemingly in a not time-related manner (Fig. 2). In the successive inoculations challenge the antimicrobial activity was not exhausted from the initial inoculum and remained efficient following a second one, albeit the second being lower in CFU as expected (Fig. 3). Finally, the abrasion test, conducted to evaluate the antimicrobial efficacy of the coating following abrasive forces, also provided a ≥ 4 log_10_ average reduction on fresh coatings (day 0) for both tested bacteria despite three abrasion cycles (Fig. 4).

### Modeled environmental experiment

**Fig. 5:**
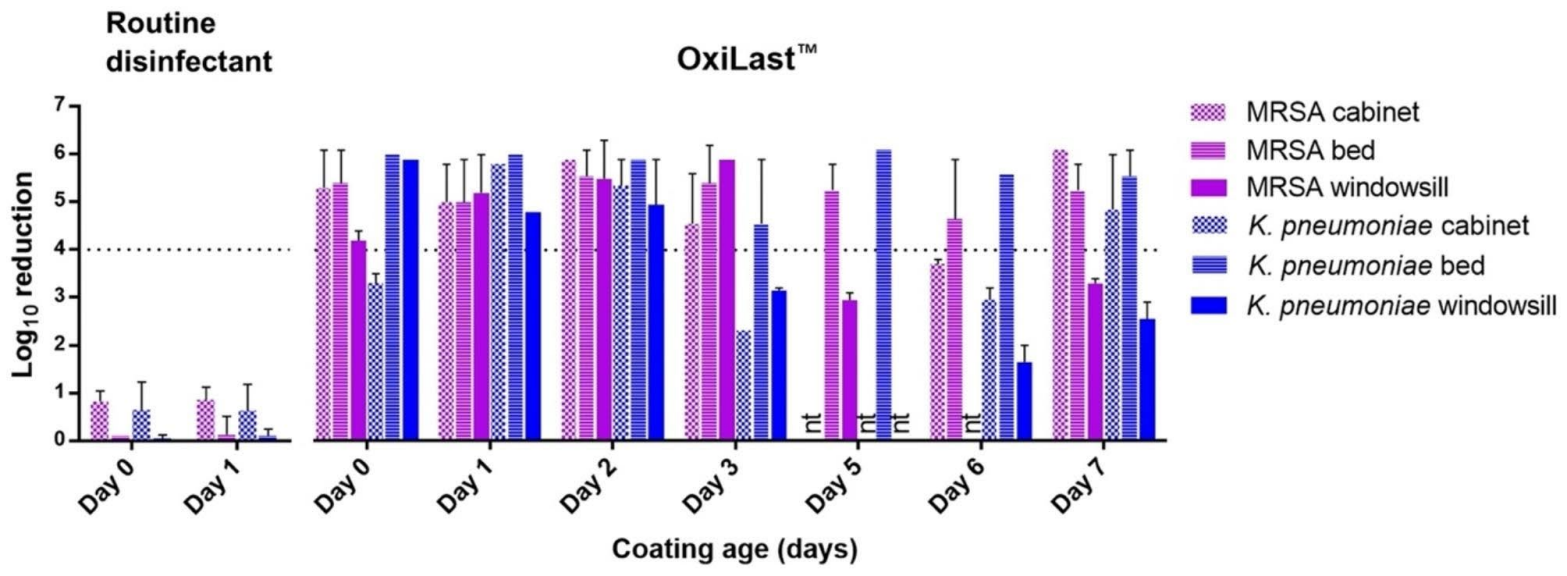
Antimicrobial activity of the OxiLast™ formulation expressed as log_10_ reduction of two tested drug-resistant ATCC reference bacterial strains inoculated onto various Oxilast-coated surfaces in a hospital room and kept for one hour contact time. The tested surfaces on the left part of the graph refer to cabinet and bedrail (only) disinfected with the routine disinfectant (KlorKleen®) and inoculated after one hour (Day 0) and 24 h (Day 1), used as a comparison. The right part of the graph shows the antimicrobial capacity of the tested formulation inoculated onto three different surfaces at various coating ages (days). The “nt” notation refers to not-tested conditions. Error bars show standard deviation

For this experimental setup, the recovery efficiency of the adopted swab method (calculated on controls) varied among the various controls; precisely, it was > 51% for the MRSA inocula and > 16% for the *K. pneumoniae*, although in 36 out of 68 inoculated controls for this whole experimental part, the recovered cell number was ≥ 6 log_10_, consistent with the inocula used. This lower efficiency with respect to the lab-phase is likely due to variations in the efficiency of cell harvesting by the swab (despite efforts to standardize its motion). Additionally, unlike the wet inocula of the laboratory phase experiment, the dry inocula used in this phase might have impacted cell viability, especially the Gram-negative *K. pneumoniae*, although this was not further investigated. While the indicated intervals depict some variations in the adopted method, these recovery efficiencies allowed for a robust calculation of the target antimicrobial activity aimed at, namely, a 4 log_10_ reduction. As expected, the standard chlorine-based disinfection solution did not provide any residual antimicrobial activity on the inocula after their application on day 0 nor day 1, showing as a proof of concept how this disinfectant, whilst suitable for disinfection from previously present bacteria on surfaces, does not retain antimicrobial activity against a subsequent contamination instance (Fig. 5, left). On the contrary, a sustained antimicrobial efficacy was observed for the OxiLast™ formulation up to seven days after its application on bedrails and cabinet surfaces, with a ≥ 4 log_10_ reduction observed for both *K. pneumoniae* and MRSA (Fig. 5, right). Fluctuations in the efficacy were nevertheless recorded in the intermediate days, where reduction of inoculated bacteria was observed as high as 6 log_10_ or down to 2.5 log_10_ (*K. pneumoniae*, Fig. 5). This variation could be due to differences in the thickness of the OxiLast™ coating (entailing a lower amount of active chlorine), which is in turn ascribable to the texture and the smoothness of the coated surfaces, or inevitable differences in the application of the coating material by the hospital staff.

**Fig. 6:**
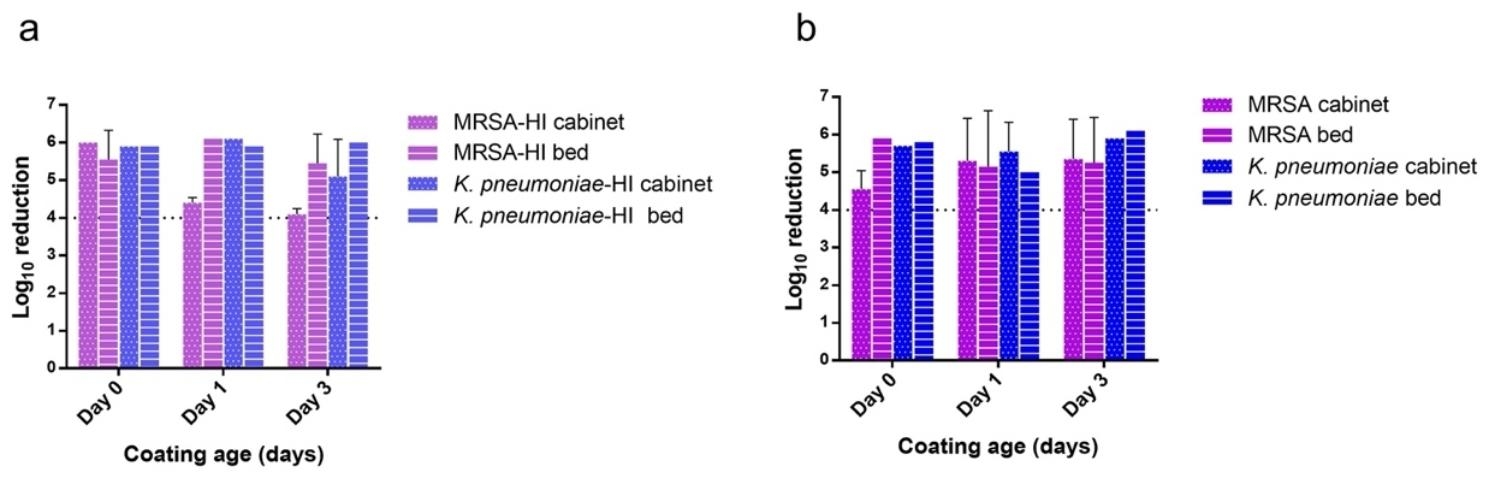
Testing of the OxiLast™ formulation onto two different coated surfaces in a hospital room at three coating ages (days). (a) test of two hospital isolates of MRSA and *K. pneumoniae* in the same conditions of the general modeled environmental testing. (b) Testing of the antimicrobial capacity of the formulation against reference ATCC strains in the presence of artificial test soil (ATS, 3 mg/ml) as an organic matrix in the tested inocula. Error bars show standard deviation

The antimicrobial effect was more consistent between the two surfaces for the MRSA, with > 3.5 log_10_ reduction on the cabinet (day 3) and both cabinets and bedrails at day 6, yet always ≥ 4 log_10_ in the other cases (Fig. 5). Such fluctuations did not seem to manifest in a time-related manner. ≥ 4 log_10_ reductions were also recorded for the two clinical isolates of MRSA and *K. pneumoniae*, tested in the same conditions as above on coatings up to three days old (Fig. 6a), showing how similar antimicrobial activity on different strains of the same tested species.

Similar to the results in the laboratory phase testing, the addition of an organic matrix (ATS solution), did not diminish the efficacy of the active chlorine in the formulation, which exerted a ≥ 4 log_10_ reduction up to day 3 in all cases (Fig. 6b).

Finally, a less sustained efficacy of the OxiLast™ formulation was observed for both species on the windowsills, for which results showed ≥ 4 log_10_ reduction up to day 2, and reduced efficacy for the following days, probably related to the smoothness of the resin painting of the surface which retained a slightly thinner OxiLast™ layer (Fig. 5, right).

## Discussion

The crucial role of environmental disinfection for the prevention of HAIs is undebated. Still, a major flaw in the current disinfection practice has to be solved, which is the window of opportunity for bacterial spread in between disinfection cycles of high-touch surfaces. Although hospital surfaces undergo routine cleaning, these surfaces will inevitably become contaminated before the next scheduled cleaning. The novel formulation of OxiLast™ was developed to support hospital hygiene by creating a robust and persistent disinfection solution applicable to hospital environmental surfaces, which would “shield” hot-spot surfaces exposed to recurrent microbial contaminations, thus curbing cross-contamination in the hospital environment. Therefore, any technological disinfection solution that works “prospectively” has important implications and deserves investigation.

In the present study, a ≥ 4 log_10_ bacterial reduction was chosen as a performance target based on the BSI-PAS-2424 2014 requirements for disinfecting agents because a minimum was not established in the case of ISO 22196:2011. It is important to contextualize (i) the choices of the CFU loads in the inocula used and (ii) the chlorine concentration of the used formulation with the broader view of the potential application of OxiLast™ in hospitals.

Human skin can harbor between roughly 10^6^ and 10^4^ bacterial cells per cm^2^, depending on the area of the body [21] which can be transferred to inert surfaces upon touch. A 10^7^ CFU inoculum is above both the ISO 22196:2011 and BSI-PAS-2424 2014 documents, which refer to 10^5^ and 10^6^ CFU on tested surfaces henceforth a ≥ 4 log_10_ reduction with this high theoretical inoculum reflects a strong disinfectant activity. A lower inoculation regime (e.g. 10^5^ CFU) would not be feasible in the present study, as it would fall right across or below the limits of detection in both sets of experiments, which in turn were dictated by both the efficiency in recovering the cells, and the fact that only a fraction of whole recovered pool of cells from the test surfaces was used (i.e. the plated fraction of the bacterial resuspension). Of note, a higher limit of detection could be achieved with a membrane filtration method, which was not employed in our experiments and deserves further study.

In order to test the effectiveness of Oxilast™, the chosen chorine concentration in the formulation for this study was 3,000 ppm, in both laboratory and hospital environment settings, whereas the routine chlorine-based solution used in the surveyed hospital is 1,000 ppm. This concentration was chosen since the desired effect is meant to be active over a prolonged time period rather than at a single point in time, thus requiring a higher load of chlorine. Nevertheless, future studies should utilize different chlorine concentrations to determine the minimally effective concentration that could be recommended for different applications.

In laboratory settings, the OxiLast™ solution demonstrated a ≥ 4 log_10_ reduction which extended for up to 7 days in 30-minute CT experiments, and similar results for most bacteria even with more practical 5-minute CT for most bacteria tested. When testing MRSA, a resilient species capable of environmental persistence, a 4 log_10_ reduction was seen for at least two days following OxiLast™ application (Fig. 2). These results were consistent also with the simulated abrasion and re-inoculation and, importantly, with an organic matrix simulant (BSA) that could compromise the effect of chlorine. In the more challenging hospital room model, a ≥ 4 log_10_ reduction was obtained until the second day on all surfaces and with all the bacteria tested. A significant bacterial reduction was seen in some experiments up to day 7, but only on bed rails and less so on other surfaces. These differential results could be due to the different textures of the surfaces, that is, a smoother surface like the resin-based paint of the windowsill would trap a thinner layer of coating, which in turn would have a less prominent effect than of a slightly rougher surface like that of the bed rails.

In the modeled environmental experiment, the consistency of the above results was also confirmed with the organic simulant (ATS) used and the two clinical strains (drug-resistant hospital isolates), and concomitantly, the non-residual antimicrobial effect of the routine disinfectant was shown. These results raise some questions on what would be the optimal use strategy for OxiLast™. An alternate-day disinfection schedule could lower disinfection costs significantly. However, since daily cleaning of visible soiling is still required for optimal disinfection and patient satisfaction, this strategy is probably not practical, and the daily disinfection schedule should remain the default with OxiLast™ as well. The benefit of such an approach could be measured by the reduction of contamination between daily cleanings in most hospital settings and reduction of disinfection in highly sensitive environments, such as intensive care units or departments accommodating immunocompromised patients, where current CDC guidelines currently recommend disinfecting twice every day. In addition, the residual effect of OxiLast™ can compensate for suboptimal or faulty daily cleaning or unavoidable gaps in cleaning, e.g. on weekends or holidays.

A different approach to residual disinfection was investigated before, taking advantage of the antimicrobial features of metals such as gold and copper. The in-vitro ability to lower bacterial counts was demonstrated many times [22], however, the clinical application of antimicrobial metal coating is complicated and expensive, as opposed to the simple addition of OxiLast™ to the existing chlorine-based solutions used currently in healthcare. A recent study by Nadimpalli et al. [23] examined a novel, quarterly ammonium-based, disinfecting agent with in vitro residual effect but failed to show a significant effect.

The combination of controlled laboratory and modeled environmental testing of OxiLast™ is an important advantage of our study, as it shows the laboratory-measured antimicrobial effects of the formulation extended to actual hospital facilities after its application by trained hospital staff personnel. The effects of disinfecting strategies and their impact in real-case scenarios in healthcare facilities have been reviewed before [24–26] and should be examined when different strategies are applied with OxiLast™. Nevertheless, one of the limitations of this part of the study is the use of only two bacterial species and the lack of real-life patient environment analysis, which might include different types of organic material in the inoculations. To try and overcome the latter limitation, we modelled these organic matrices, although further clinical-environment study is needed to assess the real-life impact of OxiLast™ fully. Altogether, with the laboratory phase, we have used only five pathogens in our study. Thus we cannot predict differences in the antimicrobial residual effect against other clinically important pathogens, although the wide spectrum of chlorine as an antimicrobial agent is universally accepted. Another limitation of our study is that in real-life conditions, the contact time between the contaminated surface and the bacteria might not be in a defined wet condition as that modeled in the laboratory phase, which in turn could impact the efficacy of the product, as observed in the differently modeled environmental experiment. In future studies, the manufacturer might need to specify a specific range of conditions based on experimental findings, including defined wet/dry CT and the corresponding antimicrobial activity claims, which should reflect operational scenarios [27]. Future studies should overcome these limitations by extending the array of tested conditions (e.g. inoculation of bacteria within different organic matrixes, such as test soils, and on different types of surfaces). Since biofilms and related extracellular matrix could hamper the efficacy of disinfectants, future studies should test the chlorine-based formulation against experimental and natural bacterial biofilms in the hospital environment [28–30]; such tests should also include a higher number of replicates than those used in the current study to enhance statistical robustness and be conducted prospectively to enable statistical modeling aimed at probing differences between organism responses.

In conclusion, OxiLast™ has shown a sustained antimicrobial effect of chlorine for up to two days following its application in the healthcare environment, with a 4 log_10_ reduction of all tested strains. This suggests an important potential for significantly reducing contamination of the patients’ environment, thus a crucial step towards reducing HAIs. Further studies are needed to establish the residual effect of OxiLast™ against other pathogens, including antibiotic susceptible bacterial species, fungi such as the emerging *Candida auris*, environmental bacteria such as *Clostridioides difficile*, or viruses, including the testing on more complicated surfaces in the healthcare environment. Moreover, future prospective studies should assess the impact of using OxiLast™ on the actual prevalence of environmental contamination in an active clinical setting and the incidence of HAI incidence over a long time.

### Electronic supplementary material

Below is the link to the electronic supplementary material.

Supplementary Material 1: Bacterial cell counts on all control surfaces

Supplementary Material 2: Schematic overview of the study plan

## Data Availability

All data analyzed in this study are presented and included in this article. Further details are available from the corresponding author.
