# Supplementary material for: Evaluation of a novel surface-coating formulation with time-extended antimicrobial activity for healthcare environment disinfection
Source: Antimicrob Resist Infect Control. 2023 Nov 23;12:133. doi: 10.1186/s13756-023-01341-w (PMC10666328; doi:10.1186/s13756-023-01341-w)
Supplement: Supplementary file 2 — Supplementary Material 2: Schematic overview of the study plan [file 13756_2023_1341_MOESM2_ESM.docx]

**Supplementary material**

**
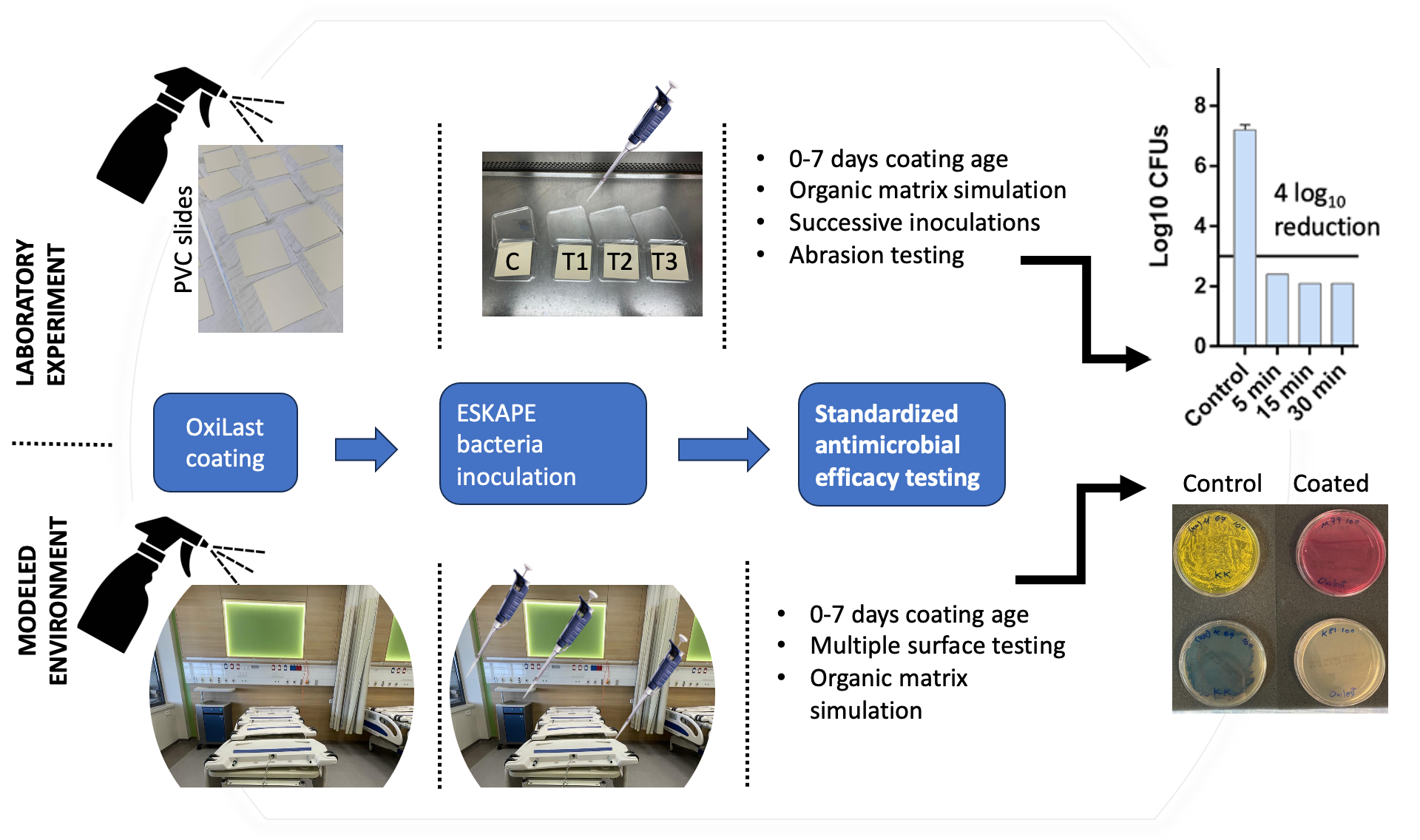
**

**Supplementary Figure 1.** Schematic overview of the laboratory phase experiment and the modeled environmental experiment phases aiming at a four log_10_ reduction of the inoculated bacterial strains onto OxiLast-coated and non-coated control surfaces.
